# The impact of anesthetic modality and cardiopulmonary bypass flow type on intraoperative intraocular pressure during coronary artery bypass grafting: A prospective, randomized controlled trial

**DOI:** 10.1097/MD.0000000000047110

**Published:** 2026-01-09

**Authors:** Dilek Uçak, Hatice Şimşek Ülkü, Emine Çiloğlu, Tolga Onur Badak

**Affiliations:** a Department of Anesthesiology and Reanimation, University of Health Sciences Adana City Training and Research Hospital, Adana, Türkiye; b Department of Ophthalmology, Adana Sevgi Eye Center Hospital, Adana, Türkiye; c Department of Cardiovascular Surgery, University of Health Sciences Adana City Training and Research Hospital, Adana, Türkiye.

**Keywords:** cardiopulmonary bypass, coronary artery bypass grafting, intraocular pressure, sevofluran, total intravenous anesthesia

## Abstract

Intraocular pressure (IOP) is an indirect marker of ocular perfusion and is critical during coronary artery bypass grafting (CABG) due to potential hemodynamic instability. The combined effects of anesthetic modality (sevoflurane vs propofol-based total intravenous anesthesia [TIVA]) and cardiopulmonary bypass (CPB) flow type (pulsatile vs nonpulsatile) on intraoperative IOP during CABG are not well established. This prospective, randomized controlled trial included 160 patients undergoing CABG, randomized into four groups (n = 40 each): Sevoflurane + pulsatile CPB (SP), sevoflurane + nonpulsatile CPB (SN), TIVA + pulsatile CPB (TP), and TIVA + nonpulsatile CPB (TN). Patients with preexisting ocular conditions were excluded. IOP was measured using a Tono-Pen Avia at four time points: before induction, after induction, after CPB initiation, and at the end of surgery. Statistical analysis included one-way analysis of variance, Tukey post hoc testing, and chi-square tests. Baseline characteristics and IOP values were comparable across groups. After induction, the TIVA groups (TP and TN) showed a significant reduction in IOP (mean difference −3.5 mm Hg; *P* < .001) compared with the sevoflurane groups. Upon CPB initiation, IOP increased in all groups, most prominently in the SN group (*P* < .01). Pulsatile flow was associated with significantly lower IOP values than nonpulsatile flow (*P* < .01), irrespective of anesthetic modality. The TP group exhibited the most stable IOP profile throughout surgery (*P* < .05). No acute glaucoma or serious ocular complications were observed. Propofol-based TIVA produced a greater reduction in IOP after induction, and pulsatile CPB contributed to a more stable intraoperative IOP profile during CABG compared with sevoflurane anesthesia and nonpulsatile CPB, respectively. Although IOP fluctuations remained within subclinical limits, these findings suggest that TIVA combined with pulsatile CPB may offer advantages in maintaining ocular hemodynamic stability, particularly in high-risk patients.

## 1. Introduction

During coronary artery bypass grafting (CABG) surgery, disruption of hemodynamic stability can significantly affect both systemic and regional perfusion. Intraocular pressure (IOP) is considered an indirect marker of ocular perfusion and is particularly critical in patients with preexisting ocular conditions such as glaucoma.^[1]^ However, the effects of CABG on IOP – particularly in relation to the anesthetic agents used and the type of cardiopulmonary bypass (CPB) flow (pulsatile or nonpulsatile) – have not been sufficiently investigated.

Volatile anesthetic agents, through their vasodilatory effects, particularly on the cerebral vasculature, can elevate intracranial pressure, which in turn may indirectly increase intraocular pressure due to the established anatomical and physiological relationship between these 2 compartments.,^[2]^ while propofol-based total intravenous anesthesia (TIVA) can reduce IOP due to its hypotensive effects.^[3]^ Similarly, pulsatile CPB has been shown to positively affect microcirculation by mimicking physiological blood flow,^[4]^ whereas nonpulsatile flow may lead to increased systemic vascular resistance and peripheral hypoperfusion.^[5,6]^

This study aims to evaluate the combined effects of different anesthetic modalities (TIVA and sevoflurane) and different CPB flow types (pulsatile and nonpulsatile) on intraoperative IOP during CABG surgery. Although visual complications are rare, fluctuations in IOP may have potential implications, especially in high-risk patients.^[7,8]^ Therefore, IOP may serve as a physiological indicator in understanding the microcirculatory impact of anesthetic and perfusion strategies used during CABG.

## 2. Materials and methods

This study was conducted as a prospective, randomized controlled clinical trial at the Cardiovascular Surgery Clinic of Adana City Training and Research Hospital, University of Health Sciences. Ethical approval was obtained from the Noninvasive Clinical Research Ethics Committee of the University of Health Sciences (Approval No: 1872, Date: 07.04.2022). Written informed consent was obtained from all participants.

### 2.1. Study population and sample size

Patients with a history of glaucoma or other ocular diseases, previous eye surgeries, contraindications to anesthetic agents, or those expected to experience severe intraoperative hemodynamic instability were excluded.

The sample size was calculated using the G Power 3.1 software. Based on preliminary data, it was determined that at least 38 patients per group would be required to detect a 3 mm Hg difference in IOP with 80% power and a 5% significance level. Accounting for a potential 5% dropout rate, 40 patients per group were included, resulting in a total sample size of 160 patients.

### 2.2. Randomization

#### 2.2.1. Patients were randomized into 4 groups

- Sevoflurane + Pulsatile CPB (SP)
- Sevoflurane + Nonpulsatile CPB (SN)
- TIVA (propofol) + Pulsatile CPB (TP)
- TIVA + Nonpulsatile CPB (TN)

Randomization was performed using computer-assisted block randomization, and group assignments were sealed in opaque envelopes to ensure allocation concealment.

#### 2.2.2. Anesthesia and surgical protocol

All patients were induced with fentanyl (2–5 µg/kg) and rocuronium (0.6 mg/kg). In the TIVA groups, induction was performed with propofol (2 mg/kg), while sevoflurane inhalation was used in the volatile groups. Maintenance was provided with continuous propofol infusion (100–200 µg/kg/min) in the TIVA groups and with sevoflurane in the volatile groups. Depth of anesthesia was monitored clinically without BIS monitoring.

CPB was initiated following systemic heparinization (ACT > 480 seconds), and the target cardiac index was maintained at 2.4 to 2.6 L/min/m².

#### 2.2.3. Intraocular pressure measurement

IOP was measured using a Tono-Pen Avia® device at 4 time points: before anesthesia induction, after induction, after initiation of CPB, and at the end of surgery. Each measurement was repeated 3 times, and the highest value was recorded. A normal IOP range of 10 to 21 mm Hg was accepted.

### 2.3. Statistical analysis

Data were analyzed using SPSS version 25.0 (Chicago). One-way ANOVA, Tukey post hoc test, and chi-square tests were employed. A *P*-value < .05 was considered statistically significant.

## 3. Results

A total of 160 patients were included in the study, with 40 patients allocated to each group. There were no significant differences among the groups in terms of age, gender, body mass index, hypertension, diabetes mellitus, or smoking status (*P* >.05, Table 1). Likewise, the average EuroSCORE II values, which assess surgical risk, were comparable across groups (SP: 2.6 ± 1.1, SN: 2.7 ± 1.3, TP: 2.5 ± 1.2, TN: 2.6 ± 1.0; *P* = .84).

**Table 1:** Baseline characteristics of study participants.

Variable	Group SP (n = 40)	Group SN (n = 40)	Group TP (n = 40)	Group TN (n = 40)	*P*-value
Age (years)	65 ± 8	66 ± 7	64 ± 9	65 ± 8	.78
Sex (Male/female)	28/12	27/13	29/11	26/14	.88
BMI (kg/m²)	27.5 ± 3.2	28.1 ± 3.5	27.3 ± 3.4	27.8 ± 3.1	.67
Hypertension (%)	25 (62.5%)	27 (67.5%)	26 (65.0%)	24 (60.0%)	.75
Diabetes mellitus (%)	15 (37.5%)	14 (35.0%)	16 (40.0%)	13 (32.5%)	.72
Smoking (%)	18 (45.0%)	20 (50.0%)	17 (42.5%)	19 (47.5%)	.69
Ejection fraction (%)	55 ± 6	54 ± 7	56 ± 5	55 ± 6	.81
Baseline IOP (mm Hg)	16.2 ± 1.8	16.5 ± 2.0	15.9 ± 1.7	16.0 ± 1.9	.82
EuroSCORE II	2.6 ± 1.1	2.7 ± 1.3	2.5 ± 1.2	2.6 ± 1.0	.84

BMI = body mass index, IOP = intraocular pressure, SN = sevoflurane + nonpulsatile cardiopulmonary bypass, SP = sevoflurane + pulsatile cardiopulmonary bypass, TN = total intravenous anesthesia + nonpulsatile cardiopulmonary bypass, TP = total intravenous anesthesia + pulsatile cardiopulmonary bypass.

Baseline intraocular pressure (IOP) values were similar among the 4 groups (SP: 15.2 ± 2.1 mm Hg, SN: 15.4 ± 2.3 mm Hg, TP: 15.1 ± 2.0 mm Hg, TN: 15.3 ± 2.2 mm Hg; *P* = .82). After induction, a significant reduction in IOP was observed in the TIVA groups (mean difference: −3.5 mm Hg, 95% CI: −4.2 to −2.8; *P* <.001, η² = 0.25). Tukey’s post hoc analysis revealed significant differences between TP and SP (*P* <.001), and between TN and SN (*P* <.001) groups.

At the initiation of CPB, IOP increased across all groups, with the most pronounced rise seen in the SN group (*P* <.01). Pulsatile flow was significantly associated with lower IOP values compared to nonpulsatile flow (*P* <.01), regardless of the anesthetic modality.

Postoperative IOP measurements showed a trend toward returning to baseline values in all groups. The TIVA + pulsatile group (TP) exhibited the most stable IOP profile throughout the surgery (*P* <.05). IOP variability was more prominent in the sevoflurane groups.

No cases of acute glaucoma, retinal ischemia, or other serious ocular complications were detected. Hemodynamic stability was maintained across all groups, with no statistically significant difference in intraoperative adverse events (*P* = .68).

**Figure 1.**
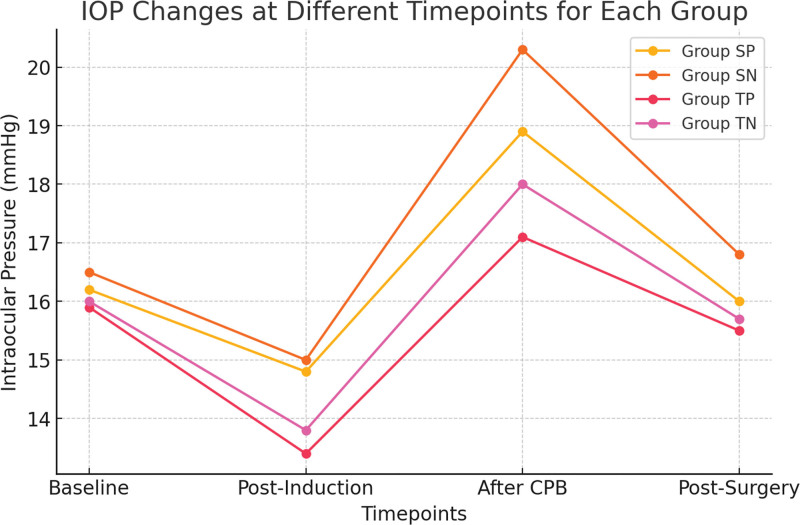
Intraoperative IOP changes across study groups. This figure illustrates intraoperative IOP changes at 4 keys surgical timepoints: Baseline (preinduction), post-induction, after CPB initiation, and post-surgery, across the study groups: SP, SN, TP, TN. TIVA-based groups (TP, TN) show a greater IOP reduction post-induction, indicating propofol’s hypotensive effect. IOP increases following CPB initiation, with nonpulsatile groups (SN, TN) exhibiting higher values, suggesting impaired ocular perfusion. The TP group maintains the most stable IOP profile, supporting its potential protective effect on ocular hemodynamics. IOP = intraocular pressure, SN = sevoflurane-nonpulsatile, SP = sevoflurane-pulsatile, TN = TIVA-nonpulsatile, TP = TIVA-pulsatile.

Subgroup analyses of IOP changes between pulsatile and nonpulsatile CPB groups are presented in Table 2, and time-dependent IOP changes are visualized in Figure 1.

**Table 2:** Intraoperative IOP changes among groups.

Timepoint	Group SP (n = 40)	Group SN (n = 40)	Group TP (n = 40)	Group TN (n = 40)	*P*-value
Baseline IOP (mm Hg)	16.2 ± 1.8	16.5 ± 2.0	15.9 ± 1.7	16.0 ± 1.9	.82
Post-induction IOP	14.8 ± 1.5	15.0 ± 1.6	13.4 ± 1.3	13.8 ± 1.4	<.01
After CPB initiation	18.9 ± 2.1	20.3 ± 2.4	17.1 ± 1.8	18.0 ± 2.0	<.01
Post-surgery IOP	16.0 ± 1.9	16.8 ± 2.1	15.5 ± 1.6	15.7 ± 1.8	.05

BMI = body mass index, CPB = cardiopulmonary bypass, IOP = intraocular pressure, SN = sevoflurane + nonpulsatile cardiopulmonary bypass, SP = sevoflurane + pulsatile cardiopulmonary bypass, TN = total intravenous anesthesia + nonpulsatile cardiopulmonary bypass, TP = total intravenous anesthesia + pulsatile cardiopulmonary bypass.

## 4. Discussion

This study is among the first randomized controlled trials to evaluate the combined effects of different anesthetic modalities (TIVA vs sevoflurane) and cardiopulmonary bypass (CPB) flow types (pulsatile vs nonpulsatile) on intraoperative intraocular pressure (IOP) during CABG surgery. According to our findings, propofol-based TIVA resulted in a more pronounced reduction in IOP compared to sevoflurane, while pulsatile CPB provided a more stable ocular hemodynamic profile by reducing fluctuations in IOP. However, despite these physiological findings, no cases of acute glaucoma, retinal ischemia, or vision loss occurred in any patient during the study. This indicates that the IOP changes observed remained within subclinical levels from a clinical perspective. Increases in IOP from 16 mm Hg to 20 mm Hg, as observed in our study, are still considered within normal physiological limits (10–21 mm Hg).^[1,7]^ Nevertheless, in individuals with ocular comorbidities such as glaucoma, such fluctuations may impact microcirculation. Therefore, in our study, IOP was considered not as a predictor of direct complications but rather as an indirect marker of ocular perfusion. Increases in IOP from 16 mm Hg to 20 mm Hg, as observed, are still considered within normal physiological limits (10–21 mm Hg).^[1]^

The IOP-lowering effect of TIVA is associated with the systemic hypotensive properties of propofol and its influence on the ciliary body.^[9,10]^ In contrast, sevoflurane has been shown to transiently increase IOP by enhancing cerebral and choroidal circulation.^[3,8]^ During CPB, nonpulsatile flow may contribute to IOP elevations due to increased peripheral vascular resistance.^[11]^ Our findings suggest that the combination of TIVA and pulsatile CPB results in the most stable IOP trajectory.

However, these findings must be interpreted within the context of certain study limitations. First, IOP measurements were performed at only 4 discrete time points rather than continuously or at shorter intervals (e.g., every 15–30 minutes). As a result, transient and short-term fluctuations in IOP may have been missed. Additionally, hemodynamic parameters such as mean arterial pressure, pump flow rate, and systemic vascular resistance during CPB were not systematically recorded, making it difficult to correlate IOP changes with systemic circulation.

Similarly, no data were available to assess the potential impact of IOP changes on renal, cerebral, or myocardial perfusion. A decrease in IOP is not always beneficial; for instance, excessively low IOP may reflect ocular hypoperfusion. Therefore, instead of focusing solely on IOP, it is recommended to evaluate it together with circulation indicators representing different organ systems.^[12,13]^

Some literature suggests that IOP variability may contribute to glaucoma progression. However, evaluating the long-term consequences of such changes during major surgeries like CABG would require larger sample sizes and extended follow-up periods. In this context, our study aims to evaluate IOP as a marker of intraoperative physiological stability rather than to provide direct clinical recommendations regarding ocular health.

Finally, agents such as dexmedetomidine, when used adjunctively with TIVA or sevoflurane, may represent a promising area for future research due to their IOP-lowering and neuroprotective properties.^[14]^ However, this agent was not used in our study; therefore, any potential effects remain hypothetical.

## Author contributions

**Conceptualization:** Dilek Uçak.

**Data curation:** Dilek Uçak, Hatice Şimşek Ülkü, Emine Çiloğlu, Tolga Onur Badak.

**Formal analysis:** Dilek Uçak, Hatice Şimşek Ülkü, Emine Çiloğlu.

**Investigation:** Dilek Uçak, Emine Çiloğlu.

**Methodology:** Dilek Uçak, Hatice Şimşek Ülkü, Emine Çiloğlu, Tolga Onur Badak.

**Project administration:** Dilek Uçak, Hatice Şimşek Ülkü, Emine Çiloğlu.

**Resources:** Dilek Uçak, Tolga Onur Badak.

**Software:** Hatice Şimşek Ülkü, Tolga Onur Badak.

**Supervision:** Dilek Uçak.

**Validation:** Emine Çiloğlu, Tolga Onur Badak.

**Visualization:** Hatice Şimşek Ülkü, Emine Çiloğlu.

**Writing – original draft:** Dilek Uçak, Emine Çiloğlu.

**Writing – review & editing:** Dilek Uçak, Hatice Şimşek Ülkü, Emine Çiloğlu.
